# The far-right and anti-vaccine attitudes: lessons from Spain’s mass COVID-19 vaccine roll-out

**DOI:** 10.1093/eurpub/ckac173

**Published:** 2023-01-19

**Authors:** Manuel Serrano-Alarcón, Yuxi Wang, Alexander Kentikelenis, Martin Mckee, David Stuckler

**Affiliations:** DONDENA Centre for Research on Social Dynamics and Public Policy, Bocconi University, Milan, Italy; DONDENA Centre for Research on Social Dynamics and Public Policy, Bocconi University, Milan, Italy; Department of Social & Political Sciences, Bocconi University, Milan, Italy; Centre for Business Research, University of Cambridge, Cambridge, UK; Department of Health Services Research and Policy, London School of Hygiene and Tropical Medicine, University of London, London, UK; DONDENA Centre for Research on Social Dynamics and Public Policy, Bocconi University, Milan, Italy; Department of Social & Political Sciences, Bocconi University, Milan, Italy

## Abstract

**Background:**

Far-right politicians in several countries have been vocal opponents of COVID-19 vaccination. But can this threaten vaccine roll-out?

**Methods:**

We take advantage of repeated cross-sectional surveys with samples of around 3800 individuals across Spain conducted monthly from December 2020 to January 2022 (*n* = 51 294) to examine any association between far-right politics and vaccine hesitancy through the whole vaccine roll-out.

**Results:**

Consistent with prior data, we found that far-right supporters were almost twice as likely to be vaccine-hesitant than the overall population in December 2020, before vaccines became available. However, with a successful vaccine roll out, this difference shrank, reaching non-significance by September 2021. From October 2021, however, vaccine hesitancy rebounded among this group at a time when the leadership of the far-right promoted a ‘freedom of choice’ discourse common among anti-vax supporters. By the latest month analysed (January 2022), far-right voters had returned to being twice as likely to be vaccine-hesitant and 7 percentage points less likely to be vaccinated than the general population.

**Conclusions:**

Our results are consistent with evidence that far-right politicians can encourage vaccine hesitancy. Nonetheless, we show that public attitudes towards vaccination are not immutable. Whereas a rapid and effective vaccine rollout can help to overcome the resistance of far-right voters to get vaccinated, they also seem to be susceptible to their party leader’s discourse on vaccines.

## Introduction

Extreme political ideologies, particularly those on the far-right, have been proposed as one of the main forces driving anti-vax attitudes, almost from the beginning of the COVID-19 pandemic.^1^^,^^2^ However, it is unclear how the relationship between far-right partisanship and vaccine attitudes evolved as vaccines became available and were successfully rolled out and whether this relationship translates into differences in vaccine uptake. Understanding these dynamics may inform policy responses designed to increase uptake in high-income countries where supply of vaccines is not a problem. For instance, as of mid-February 2022, 17% of the Spanish population was not yet fully vaccinated, while the figure was 36% in the USA.^3^

The political element of vaccine hesitancy often involves conspiracy theories and a denial of the state’s legitimacy to intervene in citizens’ health.^4^ Resistance to COVID-19 vaccines was not unexpected. Anti-vax movements are as old as vaccination. In 19th-century England saw widespread working-class opposition to compulsory smallpox vaccination,^5^ while the 1900s saw anti-vaccine riots in Brazil prompted by unfair treatment of the poor.^6^ In the early 2000s, anti-vaccine sentiment increased following Wakefield’s discredited study linking the Measles, Mumps, and Rubella (MMR) vaccine to autism, encouraged by extensive messaging on the internet.^7^ More recent anti-vax movements, often encouraged by right-wing politicians, emphasize the importance of ‘freedom of choice’ or ‘civil liberty’ as a fundamental right of citizens who oppose vaccination or any related compulsory measures.^8^

Recent academic studies have begun to investigate empirically the apparent connection between political ideology and attitudes to vaccines, and not limited to COVID-19. For example, Krupenkin^9^ found that, in the particular political context of the USA, presidential out-partisans (members of the opposition party) are less likely to believe that vaccines are safe and hence less likely to comply with government recommendations on MMR, influenza or smallpox vaccines than presidential co-partisans. This was explained in part by differences in social and institutional trust in the governing party.^9^^,^^10^

The advent of COVID-19 presents a new opportunity to examine the political determinants of vaccine hesitancy. Sharma *et al*.^1^ used Twitter data from the USA and found that far-right misinformation/conspiracy communities opposed to COVID-19 vaccines featured prominently in right-leaning accounts. Using a web-based survey, Gerretsen *et al*.^2^ show that right-wing political affiliation in the USA is a major determinants of vaccine hesitancy. In France, a representative survey during the initial phase of the pandemic (April 2020) revealed attitudes towards future COVID-19 vaccines that were significantly correlated with political partisanship.^11^ Individuals belonging to far-left and far-right parties were more vaccine-hesitant.^11^ It is important to note that the definition and ideology of far-right parties is not exactly the same across countries. They may vary in their approach to economics (pro- or anti-capitalist), social issues (conservative or libertarian) and adoption of identity politics. However, they all have in common to be at the right extreme of each country’s political spectrum.^12^

In this article, we focus on Spain, a country that has accomplished a successful vaccine rollout, despite high levels of political polarization.^13^^,^^14^ We have the following objectives: first, we examine the effect of partisan affiliation on vaccine attitudes and actual vaccine uptake. Second, we ask how this relationship evolved during the national vaccination rollout. Third, we examine whether this relationship is sensitive to the rhetoric of political leaders. To do so, we track measures of vaccine hesitancy in Spain monthly by political affiliation from December 2020, before vaccination started, up to January 2022. We find that political support for the far-right party Vox is the main determinant of vaccine hesitancy across the entire period. Over time, attitudes to vaccines among far-right voters converge with those of voters for other parties. Additionally, we document an increase in far-right voters’ vaccine hesitancy after the far-right party leader legitimizes non-vaccination as a matter of personal freedom.

Our findings make important contributions to the extant academic literature. First, we examine how the relationship between partisan affiliation and vaccine hesitancy dynamically evolves throughout the period when vaccines became available and not only at one point in time as in previous research.^2^^,^^11^ Second, we go one step further and test whether the observed relationship between political affiliation and self-declared vaccine hesitancy translates into vaccination uptake. Third, we show how politicians’ views on vaccines can influence the vaccine hesitancy of their voters, thereby presenting evidence on the mechanism of attitudinal change vis-à-vis vaccines.

### COVID-19 vaccine rollout and anti-vax attitudes in Spain

Spain participated in the joint procurement of COVID-19 vaccines with other European Union (EU) member states, within the EU vaccine strategy.^15^ The distribution of vaccines was proportional to the population of each member state. Although the vaccination rollout was decentralized to the Spanish regions, all followed common national guidelines.^16^ Vaccination started in the end of December 2020. The supply of doses was limited during the first 4 months and priority was given to care home residents, healthcare and social workers, very disabled people, essential workers and people over 80. From April 2021, as more doses became available, vaccination sped up and younger cohorts were progressively included. During the summer, vaccination opened to all age groups, with walk-in vaccination centres established in all regions. By 1 September 2021, 78% of Spain’s population was vaccinated with at least one dose (Supplementary figure A1), ranking third in Europe (after Portugal and Malta) despite the absence of a national vaccine mandate.

Spain’s population has traditionally had a high level of trust in vaccines,^15^ with vaccination rates higher than in most high-income countries. For instance, 66% of the population aged 65 and above was vaccinated against influenza in 2019, compared to OECD average of 46%. Similarly, childhood vaccination rates against measles or hepatitis B are well over 90%, despite not being mandatory.^17^ In this context, the room for anti-vax discourses to penetrate public opinion was very narrow and there is no organized anti-vax movement as seen in many other countries such as the UK, Italy or France.^18^ Nonetheless, Spain is not entirely immune to the waves of anti-vax discourses, illustrated by a diphtheria death after the parents being ‘tricked’ by anti-vaccination movements.^19^ Those individuals who actively oppose COVID-19 vaccines tend to be loosely coordinated by small professional groups, such as ‘Médicos por la Verdad’.^20^ Their discourses mainly disseminated via social media platforms.^21^

Most political parties supported the vaccination campaign and actively encouraged people to get vaccinated.^22^ This is likely a result not only of the underlying favourable public attitude to vaccines but also because vaccination was organized by the regions, with most major parties being in government in at least some regions. This could have created a virtuous vaccination race between regions, with high rates reflecting well on governing political parties. In brief, all political parties had political incentives to encourage vaccination.

The only major political party with no governing responsibilities in any region was the far-right party, Vox. Although several party members had explicitly supported vaccination on their social media accounts,^23^ others—including the leader of the party—were more ambiguous.^22^ Importantly, on September 17, the far-right leader, Santiago Abascal, refused to disclose his vaccination status on the most popular far-right radio show. Despite the insistence of the interviewer, Abascal claimed that vaccination was a matter of privacy and ‘personal freedom’ and that he would ‘not preach in favour nor against vaccination’.^23^ The show is called ‘Las mañanas de Federico’ and it is the leading programme of the radio ‘Esradio’, the 6th largest radio audience in Spain, with an average of 618 000 daily listeners.^24^ This interview lead to intense media discussion about Abascal’s vaccination status and to debate in the far-right and no-vax social media communities about freedom of choice.^25^ This spike of media attention is clearly reflected in Google search trends (Supplementary figure A2). As noted above, we are interested in the effect of such political discourses on voters’ vaccination attitudes.

### Political context in Spain: the end of bipartisanship and the rise of the far-right party

Since the 80s the Spanish political system has been characterized as an imperfect bipartisanship with alternation in power between the centre-left PSOE and the conservative PP. However, after the 2007 Great Recession, the joint support for these two parties went down to the lowest share of votes of 50% in the 2015 general elections.^26^ The main challengers to the bipartisanship were the leftist Podemos (21% of votes), and the centrist Ciudadanos (14% of votes). The rise of the far-right party Vox started later in 2018 as it got 11% of the votes in regional elections of Andalusia. Most current Vox voters used to vote for PP.^27^ In some ways, Vox can be considered a far-right splinter party from the conservative PP since it was founded by individuals who had previously been PP members. On a scale 1 (left) to 10 (right) 10, 21% VOX voters located themselves at 10, while only 7% of PP voters did so (CIS Barometer December 2020). The far-right party took 15% of the votes in the last elections of November 2019.^26^ After those elections, the central government has been run by a coalition government between the centre-left PSOE and the leftist Podemos. During our period of analysis, the voting intentions have remained relatively stable and similar to those of the last elections (Supplementary figure A3).

## Methods

### Data

We use data from the Barometers of the Centre for Sociological Research (*Centro de Investigaciones Sociologicas*—*CIS*). This is a monthly cross-sectional survey of around 3800 respondents with questions on political and social issues. The survey is representative of the population in Spain over 18 years old who have Spanish nationality (either Spanish-born or naturalized) and therefore the right to vote in the general elections. This survey has traditionally been used to measure voting intentions and attitudes towards political and social issues of the Spanish population. It also includes socioeconomic and demographic characteristics of the respondents and, since the outbreak of the epidemic, a set of COVID-19-related questions. We use data from December 2020 to January 2022 (Supplementary table A1).

We measure *vaccine hesitancy* using a dummy variable derived from the following question to those yet unvaccinated: ‘Would you be willing to vaccinate when your turn arrives?’ Those who responded ‘No’, or ‘I don’t know’ have the *vaccine hesitancy* dummy variable equal to one. The variable is equal to zero for those who are already vaccinated or declare themselves willing to get vaccinated. In a secondary analysis, we also use self-reported actual vaccination status as an outcome variable.

### Multivariate analysis

We run the following logit model separately for each of the monthly barometers: ${\mathrm{Vaccine}}_{i}$ measures vaccine hesitancy in the main analysis or actual vaccination status in the secondary analysis.


$${{\mathrm{Vaccine}}_{i}\;=\;\beta }_{0}+\;\beta_{1}{\mathrm{Voting}\;in\;\mathrm{past}\;\mathrm{elections}}_{i}{+\;X}_{i}\gamma +\;\alpha_{r}+e_{i}$$

${\mathrm{Voting}\;in\;\mathrm{past}\;\mathrm{election}}_{i}$ is a categorical variable that measures self-reported voting in the last national elections of November 2019. We codified the political parties into the following groups: left/far-left, centre-left, centre, centre-right, far-right and no voting or no response, based on the European parliament’s political groups (Supplementary table A2). The base category in the model is voting *centre*.

$X_{i}$ is a vector of control variables including age group, sex, marital status (married or not married), education level (tertiary education or lower than tertiary), employment status (employed, unemployed, retired, student, housekeeper or other), nationality (foreign nationality or not), religion (catholic, other religion, or atheist/agnostic) and two Covid-related dummies: having been diagnosed and having being hospitalized due to COVID-19. We report coefficients as marginal effects on the probability of being vaccine-hesitant (or being vaccinated in the secondary analysis). We include region fixed effects ($\alpha_{r}$) to control for unobserved factors common at the regional level such as pace of vaccine rollout or COVID-19 incidence. All analyses include survey weights provided by the CIS.

## Results

### Descriptive results

In table 1, we report the summary statistics of our survey sample from the first wave (December 2020), the last wave (January 2022) and the pooled sample. Vaccine hesitancy drastically decreased from 33% in December 2020 to only 2.4% in January 2022. On average, 25% reported voting for centre-left in the last national election, 17% for the left/far-left, 13% for the centre-right, 10% for centre and 6% for the far-right. Twenty-nine percent reported not voting or refused to disclose whom they voted for. These percentages are stable across waves. The samples are also balanced with respect to the other sociodemographic factors across time.

**Table 1:** Summary statistics

	December 2020		January 2022		Pooled sample (December 2020–January 2022)	
Variable	Mean	S.E.	Mean	S.E.	Mean	S.E.
**Vaccine hesitancy**	0.336	0.473	0.024	0.152	0.124	0.330
**Vaccinated^a^**	–	–	0.963	0.188	0.661	0.473
**Voting in last election**						
Left/far-left	0.191	0.393	0.157	0.364	0.166	0.372
Centre-left	0.237	0.426	0.243	0.429	0.253	0.435
Centre	0.113	0.317	0.082	0.275	0.096	0.295
Centre-right	0.109	0.312	0.144	0.351	0.135	0.341
Far-right	0.045	0.206	0.059	0.235	0.060	0.237
No voting or no response	0.304	0.460	0.314	0.464	0.290	0.454
**Control variables**						
Age						
18–29	0.120	0.325	0.124	0.329	0.124	0.330
30–39	0.126	0.332	0.130	0.336	0.135	0.342
40–49	0.228	0.420	0.217	0.412	0.213	0.409
50–59	0.194	0.395	0.211	0.408	0.199	0.399
60–69	0.167	0.373	0.173	0.378	0.180	0.384
70+	0.165	0.371	0.145	0.352	0.149	0.356
Men	0.484	0.500	0.514	0.500	0.491	0.500
Women	0.516	0.500	0.486	0.500	0.509	0.500
Not married	0.441	0.497	0.484	0.500	0.461	0.498
Married	0.559	0.497	0.516	0.500	0.539	0.498
Tertiary education	0.396	0.489	0.395	0.489	0.412	0.492
Lower than tertiary education	0.604	0.489	0.605	0.489	0.588	0.492
Employed	0.544	0.498	0.570	0.495	0.546	0.498
Unemployed	0.101	0.301	0.093	0.291	0.108	0.311
Retired	0.275	0.447	0.245	0.430	0.261	0.439
Student	0.042	0.200	0.046	0.209	0.042	0.200
Housekeeper	0.036	0.186	0.039	0.194	0.038	0.192
Other	0.002	0.040	0.007	0.084	0.005	0.072
Nationality						
Only Spanish	0.979	0.145	0.962	0.191	0.971	0.168
Spanish and other	0.021	0.145	0.038	0.191	0.029	0.168
Religion						
Catholic	0.599	0.490	0.591	0.492	0.594	0.491
Other religion	0.026	0.161	0.028	0.164	0.026	0.160
Atheist or agnostic	0.375	0.484	0.382	0.486	0.380	0.485
Covid						
Never diagnosed	0.966	0.181	0.848	0.359	0.923	0.267
Ever diagnosed	0.029	0.168	0.145	0.352	0.070	0.255
Ever hospitalized	0.005	0.071	0.007	0.084	0.007	0.085
Number of observations	3702		3634		51 294	

a Vaccination status was only asked from March 2021.

**Figure 1:**
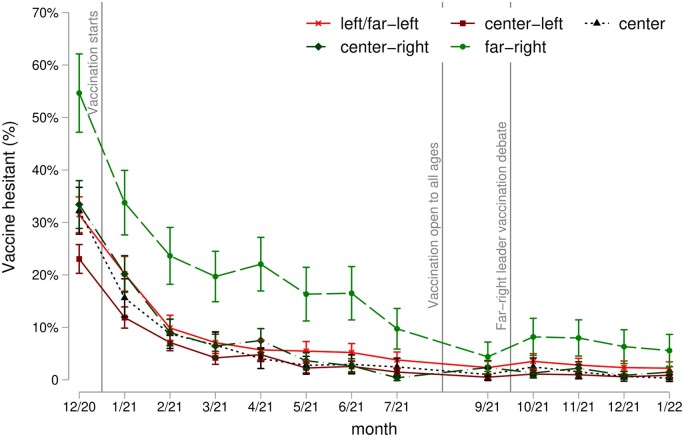
Vaccine hesitancy by vote in last elections over time

In figure 1, we show vaccine hesitancy by voting groups over time. In December 2020, far-right voters were among those most unwilling to be vaccinated, with 55% being vaccine-hesitant, compared to 23% of centre-left voters, the group with the least vaccine hesitancy. In between the two extremes were those voting left, centre and centre-right, each with a bit more than 30% of vaccine-hesitant individuals. After the vaccination programme started, vaccine hesitancy decreased for all groups and continued to fall steadily as vaccination rollout sped up. The decreasing trend in vaccine hesitancy only seems to be interrupted in April 2021, coinciding with news about blood clots linked with the Astrazeneca vaccine. Far-right voters were consistently less willing to be vaccinated until September 2021, when their attitudes converged with other voting groups. However, vaccine hesitancy among far-right voters rebounded in October following media discussion about the far-right leader’s vaccination status. This descriptive analysis might, however, be confounded by other factors that determine vaccine attitudes and vaccine access such as age or education, and that are also related to voting intentions (Supplementary table A3).

### Multivariate analysis results

**Figure 2:**
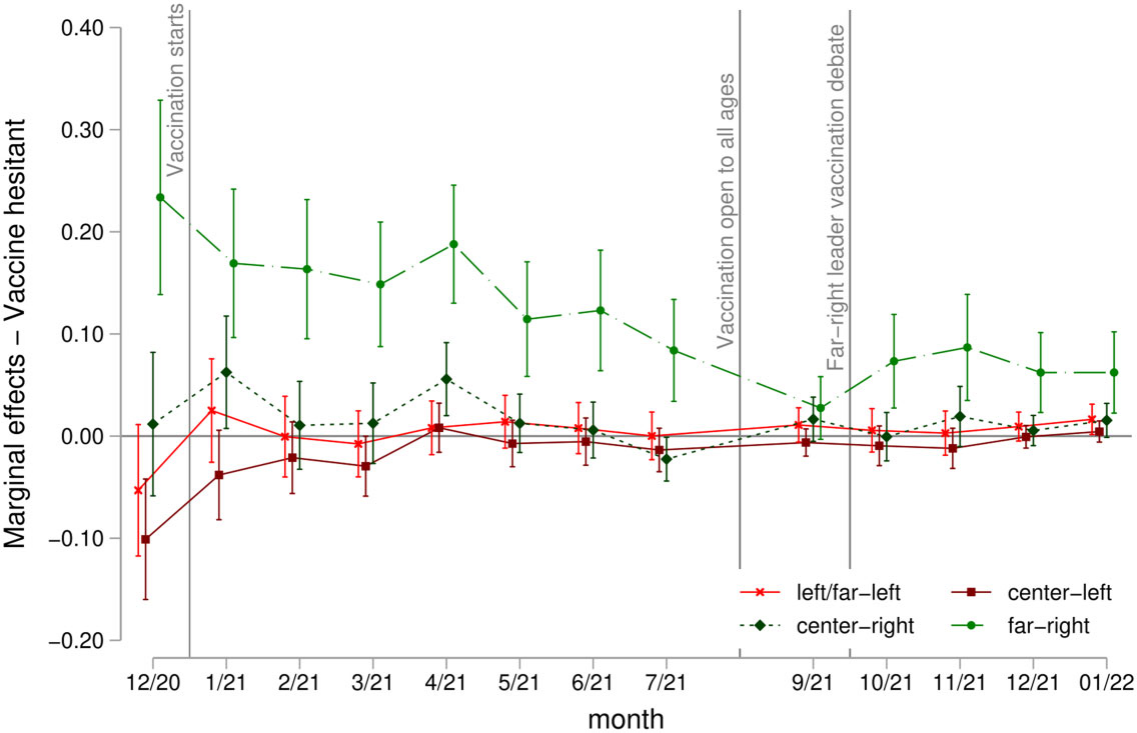
Marginal effects on the probability of being vaccine-hesitant by vote in last elections

In figure 2, we report the marginal effect on the probability of being vaccine-hesitant by voting group, after adjusting for the control variables. Far-right voters are the only group significantly more likely to be vaccine-hesitant. Before vaccination started, they were 22 percentage points (pp) more likely to be vaccine-hesitant compared to those voting centre. This difference dropped to 18 pp right after the beginning of the vaccination campaign and started to decrease more from May (11 pp) to July (8 pp) as vaccination sped up. The association between far-right voters and vaccine hesitancy became insignificant in September after vaccination opened up to the broader population, showing that the unwillingness to vaccinate among the most reluctant political group vanishes as the vaccination process advances. However, the relationship between far-right voting and vaccine hesitancy becomes significant again after the media discussion about the far-right leader’s vaccination status: far-right voters were around 7 pp more likely to be vaccine-hesitant than centrist voters. This accounts for more than 100% with respect to the population average probability of being vaccine-hesitant (Supplementary table A4). The rest of the voting groups were generally not significantly different from the centrist voters in terms of vaccine hesitancy across the vaccination period.

Regarding other factors affecting vaccine hesitancy (Supplementary table A5), women and those less educated seem to be more hesitant but only during the first months of the vaccination campaign. Adherence to any religious faith other than Catholicism is the only other factor that significantly increases vaccine hesitancy across whole vaccination process. However, this might be confounded by other cultural and social characteristics from the country of origin of those respondents who profess other religions. For instance, the second most important country of origin of the immigrant population in Spain is Romania, a country whose main religion is not catholic (Orthodox Christianity) and simultaneously have very low levels of vaccine take-up.^27^ However, we cannot further explore this hypothesis in our data since we do not have information on the country of origin nor on the specific religion for the non-catholic respondents.

**Figure 3:**
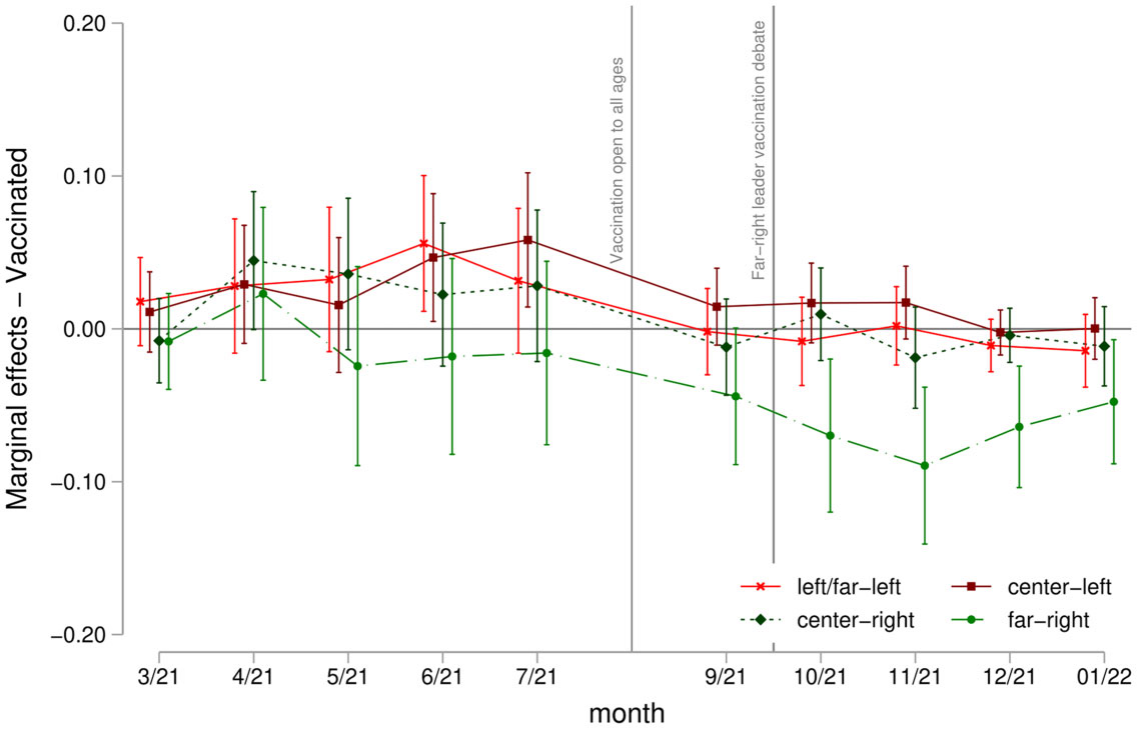
Marginal effects on the probability of being vaccinated by vote in the last election

In figure 3, we examine the relationship between voting and actual vaccine uptake. Until summer 2021, voting far-right does not appear significantly associated with actual vaccination. On the contrary, centre-left voters appear more likely to be vaccinated. However, these results might be partially confounded by the nature of the vaccination rollout, which prioritized vulnerable groups until the summer of 2021. Subsequently, when vaccination was open to the broader population, the only voting group significantly less likely to be vaccinated is far-right voters. This relationship is especially strong after the media discussion of the vaccine status of the far-right leader when far-right voting is associated with a 7–8 pp lower probability of vaccination. These results confirm that the observed relationship between far-right voting and vaccine hesitancy translates into a lower vaccine uptake.

### Robustness checks

We carry out two robustness checks of our results. First, we created a variable of political affiliation based on voting intention and political sympathy as an alternative to voting in past elections, since the latter might be affected by recall bias. This new variable is based on the party they chose in the following *voting intention* question: ‘Imagine that tomorrow there are new general elections, which party would you vote for?’ To those who declared not to vote, or ‘don’t know to’ in the previous question, we assign them to the party that they chose in the following sympathy question: ‘Would you tell me towards which party you feel more sympathy?’ Then, we group them into ideological groups following Supplementary table A2. The remaining ones were groups in the group ‘no voting or no response’. Our main results are both quantitative and qualitatively similar (Supplementary figures A4 and A5).

Second, we tested whether the observed increase in vaccine hesitancy among far-right voters after their leader’s vaccine media debate can be explained by how some regions required COVID-19 vaccination certificates to enter leisure venues around the same dates. This requirement could have created a backlash against vaccination among those who were more hesitant (i.e. far-right voters). We tested this by looking at the evolution of vaccine hesitancy among far-right voters in regions with COVID-19 vaccination certificates vs. regions without certificates (Supplementary figure A6). Far-right voters in both regions showed similar increases in vaccine hesitancy after October 2021, suggesting that the rebound in vaccine hesitancy among them is not explained by the implementation of COVID-19 vaccination certificates.

## Discussion

Our study makes several important empirical observations. We confirm prior studies indicating that far-right partisanship is a major determinant of COVID-19 vaccine hesitancy in Spain. Importantly, we move beyond these studies to demonstrate that such political resistance can be neutralized by effective vaccination campaigns. By 9 months after the campaign began, when vaccination was open to everyone, their attitudes had converged with the wider population. However, we also document a rebound in vaccine hesitancy among far-right supporters after their leader legitimized the ‘freedom of choice’ discourse of the no-vax community. This suggests that vaccine attitudes of far-right voters can be easily influenced by their leader’s views on vaccines. Consequently, convergence in vaccine attitudes with vaccination rollout should not be taken for granted.

Before interpreting these findings further, we must note several important limitations. First, ideally one would use longitudinal panel data to relate the vaccination rollout to the evolution of the individual attitudes towards vaccination. However, such data does not exist. Still, we use, to the best of our knowledge, the only dataset representative of a country that tracks political and vaccine attitudes monthly all through the COVID-19 vaccination rollout. Second, we rely on self-reported voting behaviours to designate political partisanship. This might be subject to recall bias and/or ‘hidden votes’. We partially address this issue by creating a measure of political affiliation based on voting intention and political sympathy and our main results hold in the robustness checks. However, we cannot rule out that such biases might also exist, to a lesser extent, in this alternative measure.

Our study confirms previous evidence using US online data that far-right partisanship is related to stronger vaccine hesitancy.^1^^,^^2^^,^^11^ On the other hand, unlike in France,^11^ voting for the far-left does not seem to be related with higher vaccine hesitancy in Spain. This is, however, in line with other studies showing that out-partisans are those more reluctant to be vaccinated. Far-left voters in Spain could be considered co-partisans since their largest party (Unidas Podemos) is part of the central government and six regional governments. On the contrary, the far-right party does not have any governing responsibilities anywhere in the country.

Some lessons can be learned from how the relationship between far-right partisanship and vaccine hesitancy evolved throughout the vaccination programme. First, the very high initial levels of vaccine hesitancy among far-right voters (over 50%) converged towards those of the other ideological groups as vaccination speeded up. Thus, the most reluctant segment of the population eventually got vaccinated as vaccination increased among the general population. We regard this convergence as a social norm effect in driving individual vaccine uptake behaviour.^28^ Indeed, social norms in Spain have generally accepted government rules and recommendations during the pandemic. For instance, 67.5% of the Spanish 50+ population reported always wearing a mask, compared to 47.6% in the rest of Europe.^29^ The success of the Spanish vaccination has, more generally, been explained by a set of factors including strong intergenerational family ties, the societal impact of a high death toll in the first pandemic waves, and a high level of trust in the public healthcare system.^15^^,^^18^

Second, our results show how far-right vaccine hesitancy rebounded after the party leader explicitly legitimated the ‘freedom of choice’ discourse of anti-vax supporters. This shows how an extremist political leader can influence attitudes to vaccination among their supporters. Since our results are based on pre-pandemic voting behaviour, these results are effectively driven by far-right voters following the party leader’s views, rather than anti-vax groups influencing voting intentions because of the political stand on vaccination of the party leader.

Further research may examine whether the convergence in vaccine attitudes reported here has also occurred in other countries with different progress in vaccination rollouts. It would be particularly interesting to know how far-right voters, and more generally the anti-vax group, reacted to national mandates for the unvaccinated. These policies explicitly contravene their ‘freedom of choice’ and, while mandates will certainly encourage them to get vaccinated, they might also create a backlash. In this regard, Mills and Rüttenauer^30^ found that vaccine mandates increased vaccine uptake in countries with below average uptake but had no obvious effect where it was already high or where supply was constrained.

Our results have also important policy implications for COVID-19 and other vaccination campaigns. They show that an initially high reluctance to get vaccinated by far-right supporters can be reduced if the vaccine programme is seen as successful, even where there is strong political polarization. On the other hand, we substantiate with real-world evidence previous experiments showing how political leaders can influence attitudes to vaccines, in some cases much more so than public health experts.^31^^,^^32^ In the present study, it did not matter that the politician concerned did not hold executive responsibilities. This reveals the importance of striving for cross-party agreement on messaging to maximize the outreach of vaccination campaigns.

## Supplementary Material

ckac173_Supplementary_Data

## Data Availability

The main datasets utilised in this article are publicly available at the Centre for Sociological Research (Centro de Investigaciones Sociologicas—CIS) website: https://www.cis.es/cis/opencm/EN/11_barometros/index.jsp.
