# Supplementary material for: The far-right and anti-vaccine attitudes: lessons from Spain’s mass COVID-19 vaccine roll-out
Source: Eur J Public Health. 2023 Jan 19;33(2):215–21. doi: 10.1093/eurpub/ckac173 (PMC10066477; doi:10.1093/eurpub/ckac173)
Supplement: ckac173_Supplementary_Data [file ckac173_supplementary_data.docx]

**Supplementary Material: Appendix**

Figure A1- Percentage of COVID-19 vaccinated population in Spain over time


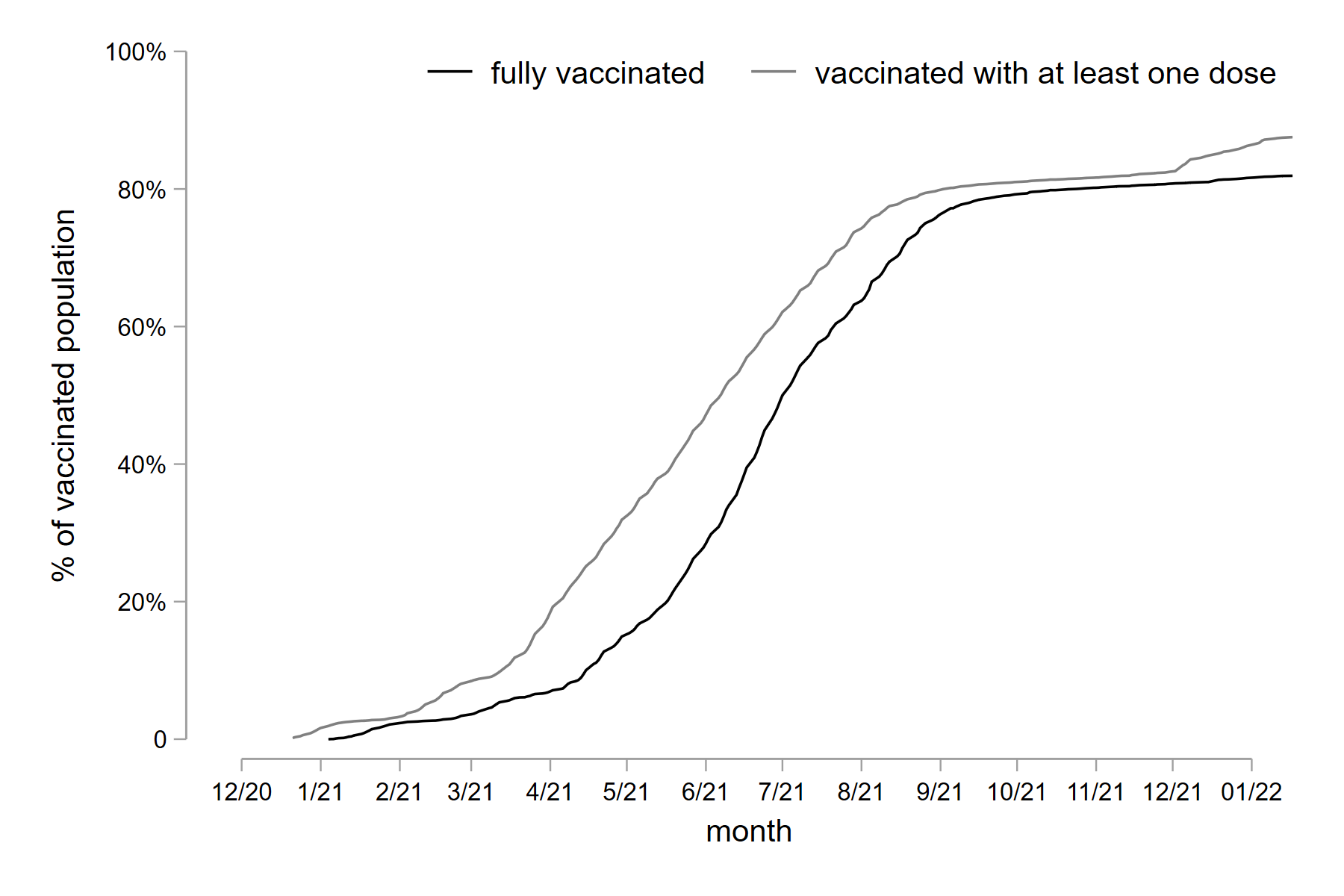


Source: Ritchie et al (2020)

Table A1 - Survey characteristics and timeline

| Month | Field days | n | n with no missing values |
| --- | --- | --- | --- |
| Dec-20 | 1 - 9 | 3817 | 3,702 |
| Jan-21 | 7 - 25 | 3862 | 3,712 |
| Feb-21 | 3 - 11 | 3869 | 3,718 |
| Mar-21 | 1 - 11 | 3820 | 3,695 |
| Apr-21 | 5 - 14 | 3823 | 3,713 |
| May-21 | 4 - 13 | 3814 | 3,689 |
| Jun-21 | 2 - 15 | 3814 | 3,667 |
| Jul-21 | 2 - 15 | 3798 | 3,660 |
| Sep-21 | 1 - 13 | 3779 | 3,635 |
| Oct-21 | 1 - 13 | 3,660 | 3,528 |
| Nov-21 | 2 - 11 | 3,779 | 3,625 |
| Dec-21 | 1 - 13 | 3,733 | 3,597 |
| Jan-22 | 3 - 14 | 3,777 | 3,634 |

Figure A2- Google searchs for "Abascal (name of the far-right party leader) vaccine"

NOTES: This figure reports the trend in Google search of the term “Abascal Vacuna” for Spain, for every week during the last 12 months. Source <https://trends.google.com/trends/?geo=ES> (data downloaded as 04/02/2022). “Abascal” is the surname of the far-right leader, “vacuna” means vaccine in Spanish. Google search trends are normalised from 0 to 100 (highest search interest) 100 is assigned to the point in time when the search term reached the highest proportion of searches relative to the total searches done at the same point of time. Search terms with low volume at a particular period of time appear as “0”. For more information on how to interpret Google search trends, see <https://support.google.com/trends/answer/4365533?hl=en>

Figure A3 – Voting intention during our period of analysis


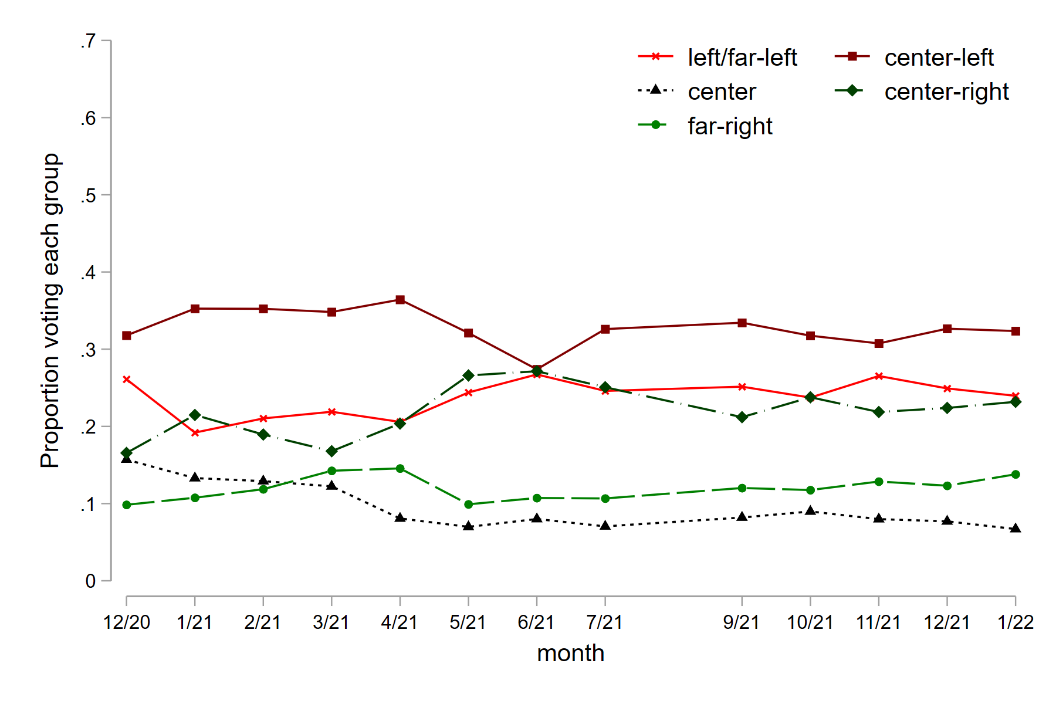


NOTES: Voting intentions among the subsample of respondents who declared their voting intentions of the CIS Barometer from December 2020 to January 2022 (n= 34,667). We classify the parties by voting groups as explained in Table A2 below.

Table A2- Classification of political parties per voting group.

| **Voting group** | **Political party** (number of observation in parenthesis) |
| --- | --- |
| Left/far-left | Unidas Podemos (4,305), ERC (1,188), En Comu Podem (419), Mas Pais (352), EH Bildu (316), En Común-Unidas Podem (246), BNG (239), Més Compromís (218) ,BNG (239) Més Compromís (218), CUP (186), Mas Pais (352) |
| Center-left | PSOE (12,048), PRC (62), Teruel Existe (23) |
| Center | Ciudadanos (3,363), JxCat(576), EAJ-PNV (494), CC-NC (84) |
| Center-right | PP (6,427), Navarra Suma (135) |
| Far-right | Vox (2,853) |
| No voting or no response | did not vote (6,428), does not answer (3,839), does not remember (1,681), voted blank (1,067), voted null (412), voted for other parties (339). |

NOTES: Authors’ classification based on the European parliament’s political groups. *Left/far-left*: The Left in the European Parliament (GUE-NGL) and Greens-European Free Alliance (Greens-EFA); *centre-left*: Progressive Alliance of Socialists and Democrats (S&D): *centre*: Renew Europe (Renew); centre*-*right: European People’s Party (EPP); *far-right*: European Conservatives and Reformists (ECR).

Table A3 – Summary statistics by voting group

|  | left/far-left | | Center-left | | Center | | Center-right | | far-right | | no vote/response | |
| --- | --- | --- | --- | --- | --- | --- | --- | --- | --- | --- | --- | --- |
| Variable | Mean | S.E. | Mean | S.E. | Mean | S.E. | Mean | S.E. | Mean | S.E. | Mean | S.E. |
|  |  |  |  |  |  |  |  |  |  |  |  |  |
| Age: 18-29 | 0.166 | 0.372 | 0.087 | 0.282 | 0.091 | 0.288 | 0.068 | 0.251 | 0.108 | 0.310 | 0.165 | 0.371 |
| Age: 30-39 | 0.191 | 0.393 | 0.105 | 0.306 | 0.142 | 0.349 | 0.093 | 0.291 | 0.198 | 0.398 | 0.127 | 0.333 |
| Age: 40-49 | 0.231 | 0.421 | 0.191 | 0.393 | 0.270 | 0.444 | 0.185 | 0.388 | 0.288 | 0.453 | 0.189 | 0.392 |
| Age: 50-59 | 0.182 | 0.386 | 0.207 | 0.405 | 0.225 | 0.417 | 0.203 | 0.402 | 0.190 | 0.392 | 0.186 | 0.389 |
| Age: 60-69 | 0.160 | 0.366 | 0.228 | 0.419 | 0.157 | 0.364 | 0.191 | 0.393 | 0.144 | 0.351 | 0.160 | 0.367 |
| Age: 70+ | 0.071 | 0.257 | 0.183 | 0.386 | 0.115 | 0.319 | 0.261 | 0.439 | 0.073 | 0.261 | 0.173 | 0.378 |
|  |  |  |  |  |  |  |  |  |  |  |  |  |
| Men | 0.534 | 0.499 | 0.449 | 0.497 | 0.533 | 0.499 | 0.486 | 0.500 | 0.643 | 0.479 | 0.454 | 0.498 |
| Women | 0.466 | 0.499 | 0.551 | 0.497 | 0.467 | 0.499 | 0.514 | 0.500 | 0.357 | 0.479 | 0.546 | 0.498 |
|  |  |  |  |  |  |  |  |  |  |  |  |  |
| Not married | 0.549 | 0.498 | 0.424 | 0.494 | 0.382 | 0.486 | 0.371 | 0.483 | 0.432 | 0.495 | 0.521 | 0.500 |
| Married | 0.451 | 0.498 | 0.576 | 0.494 | 0.618 | 0.486 | 0.629 | 0.483 | 0.568 | 0.495 | 0.479 | 0.500 |
|  |  |  |  |  |  |  |  |  |  |  |  |  |
| Tertiary education | 0.504 | 0.500 | 0.378 | 0.485 | 0.540 | 0.498 | 0.438 | 0.496 | 0.375 | 0.484 | 0.341 | 0.474 |
| Lower than tertiary education | 0.496 | 0.500 | 0.622 | 0.485 | 0.460 | 0.498 | 0.562 | 0.496 | 0.625 | 0.484 | 0.659 | 0.474 |
|  |  |  |  |  |  |  |  |  |  |  |  |  |
| Employed | 0.638 | 0.481 | 0.490 | 0.500 | 0.656 | 0.475 | 0.485 | 0.500 | 0.656 | 0.475 | 0.483 | 0.500 |
| Unemployed | 0.111 | 0.314 | 0.102 | 0.303 | 0.079 | 0.269 | 0.074 | 0.263 | 0.114 | 0.317 | 0.133 | 0.340 |
| Retired | 0.180 | 0.385 | 0.328 | 0.469 | 0.209 | 0.407 | 0.361 | 0.480 | 0.165 | 0.371 | 0.270 | 0.444 |
| Student | 0.051 | 0.220 | 0.028 | 0.165 | 0.027 | 0.163 | 0.020 | 0.140 | 0.026 | 0.159 | 0.063 | 0.243 |
| Housekeeper | 0.017 | 0.128 | 0.047 | 0.211 | 0.025 | 0.157 | 0.054 | 0.226 | 0.033 | 0.177 | 0.044 | 0.205 |
| Other | 0.003 | 0.057 | 0.005 | 0.073 | 0.003 | 0.056 | 0.005 | 0.073 | 0.006 | 0.080 | 0.007 | 0.084 |
|  |  |  |  |  |  |  |  |  |  |  |  |  |
| Nationality: only Spanish | 0.981 | 0.137 | 0.971 | 0.167 | 0.985 | 0.123 | 0.980 | 0.140 | 0.984 | 0.125 | 0.956 | 0.206 |
| Nationality: Spanish and other | 0.019 | 0.137 | 0.029 | 0.167 | 0.015 | 0.123 | 0.020 | 0.140 | 0.016 | 0.125 | 0.044 | 0.206 |
|  |  |  |  |  |  |  |  |  |  |  |  |  |
| Table continues in next page… |  |  |  |  |  |  |  |  |  |  |  |  |
| Religion - Catholic | 0.252 | 0.434 | 0.586 | 0.493 | 0.664 | 0.472 | 0.876 | 0.330 | 0.784 | 0.412 | 0.614 | 0.487 |
| Religion - Other religion | 0.017 | 0.128 | 0.025 | 0.155 | 0.015 | 0.122 | 0.014 | 0.116 | 0.018 | 0.132 | 0.045 | 0.208 |
| Religion - Atheist or agnostic | 0.732 | 0.443 | 0.389 | 0.488 | 0.321 | 0.467 | 0.110 | 0.313 | 0.198 | 0.399 | 0.341 | 0.474 |
|  |  |  |  |  |  |  |  |  |  |  |  |  |
| Covid - Never diagnosed | 0.927 | 0.261 | 0.927 | 0.260 | 0.919 | 0.273 | 0.922 | 0.268 | 0.916 | 0.278 | 0.922 | 0.268 |
| Covid - Ever diagnosed | 0.067 | 0.251 | 0.065 | 0.247 | 0.075 | 0.263 | 0.068 | 0.252 | 0.077 | 0.266 | 0.071 | 0.257 |
| Covid - Ever hospitalised | 0.006 | 0.077 | 0.007 | 0.085 | 0.007 | 0.082 | 0.010 | 0.099 | 0.007 | 0.086 | 0.007 | 0.085 |
|  |  |  |  |  |  |  |  |  |  |  |  |  |
| Number of observations | 8515 | | 12984 | | 4945 | | 6910 | | 3053 | | 14887 | |

NOTES: Pooled sample (December 2020 – January 2022)

Table A4 - Proportion of respondents that are vaccine hesitant and vaccinated per month

|  | Vaccine hesitancy | | Vaccinated | |
| --- | --- | --- | --- | --- |
| Month | Mean | S.E. | Mean | S.E. |
| Dec-20 | 0.337 | 0.008 |  |  |
| Jan-21 | 0.209 | 0.007 |  |  |
| Feb-21 | 0.119 | 0.005 |  |  |
| Mar-21 | 0.091 | 0.005 | 0.052 | 0.004 |
| Apr-21 | 0.085 | 0.005 | 0.15 | 0.006 |
| May-21 | 0.06 | 0.004 | 0.379 | 0.008 |
| Jun-21 | 0.054 | 0.004 | 0.554 | 0.008 |
| Jul-21 | 0.037 | 0.003 | 0.758 | 0.007 |
| Sep-21 | 0.025 | 0.003 | 0.946 | 0.004 |
| Oct-21 | 0.031 | 0.003 | 0.951 | 0.004 |
| Nov-21 | 0.03 | 0.003 | 0.953 | 0.003 |
| Dec-21 | 0.023 | 0.002 | 0.967 | 0.003 |
| Jan-22 | 0.024 | 0.002 | 0.964 | 0.003 |

Table A5 - Logit model on the probability of vaccine hesitancy. Marginal effects

|  | (1) | (2) | (3) | (4) | (5) | (6) | (7) | (8) | (9) | (10) | (11) | (12) | (13) |
| --- | --- | --- | --- | --- | --- | --- | --- | --- | --- | --- | --- | --- | --- |
| VARIABLES | 12/20 | 01/21 | 02/21 | 03/21 | 04/21 | 05/21 | 06/21 | 07/21 | 09/21 | 10/21 | 11/21 | 12/21 | 01/22 |
|  |  |  |  |  |  |  |  |  |  |  |  |  |  |
| Voting in last election |  |  |  |  |  |  |  |  |  |  |  |  |  |
| (ref.: centre) |  |  |  |  |  |  |  |  |  |  |  |  |  |
| left/far-left | -0.053 | 0.025 | -0.001 | -0.008 | 0.008 | 0.014 | 0.008 | 0.000 | 0.011 | 0.006 | 0.003 | 0.009 | 0.016** |
|  | (0.033) | (0.026) | (0.020) | (0.016) | (0.013) | (0.013) | (0.013) | (0.012) | (0.009) | (0.011) | (0.011) | (0.007) | (0.008) |
| centre-left | -0.101*** | -0.038* | -0.021 | -0.029** | 0.008 | -0.007 | -0.005 | -0.014 | -0.006 | -0.009 | -0.012 | -0.001 | 0.004 |
|  | (0.030) | (0.022) | (0.018) | (0.015) | (0.012) | (0.012) | (0.012) | (0.011) | (0.007) | (0.010) | (0.010) | (0.006) | (0.005) |
| centre-right | 0.012 | 0.062** | 0.011 | 0.013 | 0.056*** | 0.013 | 0.006 | -0.023** | 0.017 | -0.001 | 0.019 | 0.005 | 0.015* |
|  | (0.036) | (0.028) | (0.022) | (0.020) | (0.018) | (0.015) | (0.014) | (0.011) | (0.011) | (0.012) | (0.015) | (0.008) | (0.008) |
| far-right | 0.234*** | 0.169*** | 0.163*** | 0.149*** | 0.188*** | 0.115*** | 0.123*** | 0.084*** | 0.028* | 0.073*** | 0.087*** | 0.062*** | 0.062*** |
|  | (0.049) | (0.037) | (0.035) | (0.031) | (0.029) | (0.029) | (0.030) | (0.025) | (0.016) | (0.023) | (0.026) | (0.020) | (0.020) |
| No voting/no response | 0.062** | 0.105*** | 0.062*** | 0.063*** | 0.070*** | 0.060*** | 0.036*** | 0.030** | 0.027*** | 0.019* | 0.026** | 0.032*** | 0.032*** |
|  | (0.030) | (0.024) | (0.019) | (0.017) | (0.014) | (0.014) | (0.013) | (0.012) | (0.009) | (0.011) | (0.012) | (0.008) | (0.007) |
| Age groups |  |  |  |  |  |  |  |  |  |  |  |  |  |
| (ref: Age 70+) |  |  |  |  |  |  |  |  |  |  |  |  |  |
| age 18-29 | 0.058 | 0.022 | 0.011 | 0.064** | 0.036 | 0.083*** | 0.064*** | 0.022 | 0.005 | 0.004 | -0.035* | -0.008 | -0.005 |
|  | (0.051) | (0.042) | (0.030) | (0.029) | (0.029) | (0.019) | (0.018) | (0.019) | (0.010) | (0.016) | (0.019) | (0.016) | (0.010) |
| age 30-39 | 0.106** | 0.059 | 0.017 | 0.028 | 0.028 | 0.075*** | 0.055*** | 0.013 | 0.015 | 0.000 | -0.015 | 0.000 | 0.014 |
|  | (0.048) | (0.040) | (0.027) | (0.024) | (0.026) | (0.018) | (0.014) | (0.016) | (0.012) | (0.014) | (0.021) | (0.016) | (0.012) |
| age 40-49 | 0.034 | 0.006 | 0.002 | 0.031 | 0.011 | 0.064*** | 0.045*** | 0.005 | 0.009 | 0.013 | -0.005 | -0.003 | 0.024** |
|  | (0.043) | (0.036) | (0.025) | (0.023) | (0.023) | (0.015) | (0.012) | (0.015) | (0.009) | (0.014) | (0.021) | (0.015) | (0.012) |
| age 50-59 | 0.005 | -0.000 | -0.018 | 0.014 | -0.009 | 0.040*** | 0.025** | 0.011 | 0.009 | -0.001 | -0.018 | -0.002 | 0.018* |
|  | (0.042) | (0.036) | (0.024) | (0.021) | (0.022) | (0.012) | (0.010) | (0.014) | (0.008) | (0.013) | (0.020) | (0.015) | (0.011) |
| age 60-69 | -0.035 | -0.030 | -0.013 | -0.002 | 0.001 | 0.019** | 0.027*** | 0.002 | -0.001 | 0.007 | -0.003 | -0.007 | 0.010 |
|  | (0.031) | (0.027) | (0.020) | (0.016) | (0.018) | (0.009) | (0.010) | (0.011) | (0.008) | (0.012) | (0.016) | (0.013) | (0.009) |
| female | 0.075*** | 0.055*** | 0.028*** | 0.021** | 0.010 | -0.001 | 0.003 | 0.001 | -0.009* | 0.000 | -0.006 | -0.005 | 0.006 |
|  | (0.017) | (0.014) | (0.011) | (0.010) | (0.009) | (0.008) | (0.008) | (0.006) | (0.005) | (0.006) | (0.006) | (0.005) | (0.006) |
| married | -0.029 | -0.056*** | -0.028** | -0.021* | -0.019* | -0.028*** | -0.025*** | -0.021*** | -0.008 | -0.010 | -0.022*** | -0.012** | -0.007 |
|  | (0.019) | (0.014) | (0.012) | (0.011) | (0.010) | (0.010) | (0.009) | (0.008) | (0.006) | (0.007) | (0.007) | (0.005) | (0.006) |
| Secondary or lower education | 0.047*** | 0.036** | 0.045*** | 0.031*** | 0.025** | 0.027*** | 0.009 | 0.000 | 0.004 | 0.011* | 0.014* | 0.006 | 0.009 |
|  | (0.018) | (0.014) | (0.012) | (0.010) | (0.010) | (0.009) | (0.008) | (0.007) | (0.006) | (0.006) | (0.007) | (0.006) | (0.007) |
| Table continues in next page….. | | |  |  |  |  |  |  |  |  |  |  |  |
| Employment status |  |  |  |  |  |  |  |  |  |  |  |  |  |
| (ref: employed) |  |  |  |  |  |  |  |  |  |  |  |  |  |
| unemployed | -0.005 | 0.022 | 0.009 | 0.018 | 0.050*** | 0.009 | 0.032** | 0.030** | 0.000 | -0.003 | 0.011 | 0.004 | 0.025** |
|  | (0.028) | (0.023) | (0.017) | (0.015) | (0.017) | (0.012) | (0.014) | (0.014) | (0.008) | (0.009) | (0.012) | (0.009) | (0.011) |
| retired | -0.062* | -0.056** | -0.011 | -0.002 | -0.030* | -0.003 | -0.007 | 0.004 | -0.001 | -0.004 | -0.016 | -0.008 | 0.010 |
|  | (0.036) | (0.028) | (0.019) | (0.021) | (0.016) | (0.021) | (0.013) | (0.015) | (0.008) | (0.011) | (0.011) | (0.009) | (0.009) |
| student | -0.035 | -0.045 | -0.045** | -0.045*** | -0.048*** | -0.017 | -0.034*** | -0.007 | -0.013 | -0.012 |  | -0.017** | 0.027 |
|  | (0.048) | (0.033) | (0.022) | (0.016) | (0.015) | (0.014) | (0.009) | (0.011) | (0.008) | (0.013) |  | (0.008) | (0.028) |
| housekeeper | -0.001 | -0.032 | 0.044 | 0.036 | 0.015 | -0.002 | 0.006 | 0.038 | 0.024 | 0.007 | -0.013 | -0.008 | 0.028 |
|  | (0.048) | (0.039) | (0.032) | (0.030) | (0.030) | (0.027) | (0.025) | (0.031) | (0.024) | (0.022) | (0.018) | (0.013) | (0.020) |
| other | 0.366* | 0.161 | 0.222 | -0.008 | -0.051 |  | 0.079 | 0.001 | 0.031 | 0.083 | 0.015 | 0.039 | 0.008 |
|  | (0.190) | (0.179) | (0.159) | (0.051) | (0.036) |  | (0.080) | (0.034) | (0.047) | (0.064) | (0.038) | (0.048) | (0.026) |
| foreign nationality | 0.014 | 0.054 | 0.019 | 0.032 | 0.004 | 0.002 | 0.007 | 0.016 | 0.007 | 0.016 | 0.015 | 0.006 | 0.004 |
|  | (0.058) | (0.040) | (0.030) | (0.023) | (0.024) | (0.021) | (0.018) | (0.013) | (0.010) | (0.014) | (0.012) | (0.011) | (0.012) |
| Religion |  |  |  |  |  |  |  |  |  |  |  |  |  |
| (ref: catholic) |  |  |  |  |  |  |  |  |  |  |  |  |  |
| other religion | 0.084 | 0.205*** | 0.110*** | 0.114*** | 0.203*** | 0.120*** | 0.032 | 0.042* | 0.086*** | 0.074*** | 0.123*** | 0.055** | 0.032 |
|  | (0.058) | (0.053) | (0.041) | (0.041) | (0.048) | (0.034) | (0.024) | (0.024) | (0.032) | (0.027) | (0.043) | (0.026) | (0.020) |
| atheist/agnostic | -0.031 | -0.015 | 0.007 | 0.026** | 0.016 | 0.001 | -0.001 | 0.017** | 0.006 | 0.024*** | 0.023*** | 0.013** | 0.014* |
|  | (0.020) | (0.016) | (0.013) | (0.012) | (0.011) | (0.009) | (0.009) | (0.007) | (0.006) | (0.007) | (0.008) | (0.006) | (0.007) |
| Diagnosed COVID-19 | 0.057 | -0.028 | -0.029 | -0.047*** | -0.007 | -0.021* | -0.007 | -0.011 | 0.014 | 0.011 | 0.009 | -0.006 | 0.007 |
|  | (0.051) | (0.029) | (0.019) | (0.015) | (0.017) | (0.012) | (0.013) | (0.010) | (0.010) | (0.012) | (0.012) | (0.008) | (0.009) |
| Hospitalised COVID-19 | -0.025 | -0.145*** | - | -0.028 | 0.023 | - | -0.002 | -0.004 | - | - | 0.015 | 0.003 | 0.015 |
|  | (0.118) | (0.056) |  | (0.048) | (0.061) |  | (0.046) | (0.032) |  |  | (0.043) | (0.027) | (0.038) |
| Region FE | Yes | Yes | Yes | Yes | Yes | Yes | Yes | Yes | Yes | Yes | Yes | Yes | Yes |
|  |  |  |  |  |  |  |  |  |  |  |  |  |  |
| Observations | 3,702 | 3,712 | 3,701 | 3,695 | 3,702 | 3,642 | 3,659 | 3,660 | 3,589 | 3,494 | 3,283 | 3,587 | 3,545 |
| NOTES: Each column reports coefficients from a different logit regression where the dependent variable is vaccine hesitancy at each month. Robust standard errors in parentheses. *** p<0.01, ** p<0.05, * p<0.1. | | | | | | | | | | | | | |

Table A6- Logit model on the probability of actual vaccination. Marginal effects

|  | (1) | (2) | (3) | (4) | (5) | (6) | (7) | (8) | (9) | (10) |
| --- | --- | --- | --- | --- | --- | --- | --- | --- | --- | --- |
| VARIABLES | 03/21 | 04/21 | 05/21 | 06/21 | 07/21 | 09/21 | 10/21 | 11/21 | 12/21 | 01/22 |
|  |  |  |  |  |  |  |  |  |  |  |
| Voting in last election |  |  |  |  |  |  |  |  |  |  |
| (ref.: centre) |  |  |  |  |  |  |  |  |  |  |
| left/far-left | 0.018 | 0.028 | 0.032 | 0.056** | 0.031 | -0.002 | -0.008 | 0.002 | -0.011 | -0.014 |
|  | (0.015) | (0.022) | (0.024) | (0.023) | (0.024) | (0.014) | (0.015) | (0.013) | (0.009) | (0.012) |
| centre-left | 0.011 | 0.029 | 0.016 | 0.047** | 0.058*** | 0.015 | 0.017 | 0.017 | -0.002 | 0.000 |
|  | (0.013) | (0.020) | (0.023) | (0.021) | (0.022) | (0.013) | (0.013) | (0.012) | (0.007) | (0.010) |
| centre-right | -0.008 | 0.045* | 0.036 | 0.022 | 0.028 | -0.012 | 0.010 | -0.019 | -0.004 | -0.011 |
|  | (0.014) | (0.023) | (0.025) | (0.024) | (0.025) | (0.016) | (0.015) | (0.017) | (0.009) | (0.013) |
| far-right | -0.008 | 0.023 | -0.024 | -0.018 | -0.016 | -0.044* | -0.070*** | -0.089*** | -0.064*** | -0.048** |
|  | (0.016) | (0.029) | (0.033) | (0.033) | (0.031) | (0.023) | (0.026) | (0.026) | (0.020) | (0.021) |
| No voting/no response | -0.002 | -0.005 | 0.002 | 0.010 | -0.001 | -0.028** | -0.018 | -0.020 | -0.045*** | -0.031*** |
|  | (0.013) | (0.019) | (0.022) | (0.021) | (0.023) | (0.014) | (0.014) | (0.013) | (0.009) | (0.011) |
| Age groups |  |  |  |  |  |  |  |  |  |  |
| (ref: Age 70+) |  |  |  |  |  |  |  |  |  |  |
| age 18-29 | -0.037 | -0.247*** | -0.803*** | -0.771*** | -0.677*** | -0.054*** | -0.020 | 0.020 | -0.003 | -0.026* |
|  | (0.031) | (0.041) | (0.025) | (0.030) | (0.037) | (0.018) | (0.018) | (0.026) | (0.016) | (0.015) |
| age 30-39 | -0.036 | -0.281*** | -0.809*** | -0.807*** | -0.421*** | -0.050*** | -0.036** | 0.016 | -0.014 | -0.024* |
|  | (0.030) | (0.038) | (0.020) | (0.023) | (0.031) | (0.016) | (0.018) | (0.025) | (0.016) | (0.013) |
| age 40-49 | -0.039 | -0.264*** | -0.816*** | -0.740*** | -0.117*** | -0.028** | -0.025 | 0.017 | -0.013 | -0.032*** |
|  | (0.030) | (0.037) | (0.019) | (0.022) | (0.021) | (0.013) | (0.016) | (0.024) | (0.015) | (0.012) |
| age 50-59 | -0.035 | -0.256*** | -0.763*** | -0.199*** | -0.047*** | -0.027** | -0.003 | 0.035 | -0.009 | -0.026** |
|  | (0.030) | (0.037) | (0.020) | (0.021) | (0.017) | (0.013) | (0.014) | (0.023) | (0.015) | (0.011) |
| age 60-69 | -0.051** | -0.104*** | -0.211*** | -0.085*** | -0.047*** | -0.016 | -0.005 | 0.021 | -0.002 | -0.012 |
|  | (0.025) | (0.032) | (0.021) | (0.016) | (0.016) | (0.011) | (0.013) | (0.019) | (0.011) | (0.009) |
| female | 0.024*** | 0.042*** | 0.067*** | 0.030** | 0.009 | 0.016** | 0.008 | 0.012* | 0.012** | 0.002 |
|  | (0.008) | (0.012) | (0.012) | (0.012) | (0.011) | (0.008) | (0.008) | (0.007) | (0.006) | (0.007) |
| married | -0.012 | -0.012 | 0.004 | 0.059*** | 0.066*** | 0.022** | 0.024*** | 0.026*** | 0.019*** | 0.007 |
|  | (0.008) | (0.013) | (0.013) | (0.013) | (0.012) | (0.009) | (0.008) | (0.009) | (0.006) | (0.008) |
| Secondary or lower education | -0.025*** | -0.051*** | -0.054*** | -0.039*** | -0.019 | -0.011 | -0.016** | -0.025*** | -0.006 | -0.015** |
|  | (0.008) | (0.012) | (0.013) | (0.012) | (0.012) | (0.008) | (0.008) | (0.008) | (0.007) | (0.008) |
| Table continues in next page… |  |  |  |  |  |  |  |  |  |  |
| Employment status |  |  |  |  |  |  |  |  |  |  |
| (ref: employed) |  |  |  |  |  |  |  |  |  |  |
| unemployed | -0.066*** | -0.142*** | -0.113*** | -0.121*** | -0.073*** | -0.018 | -0.012 | -0.021 | -0.018 | -0.031** |
|  | (0.010) | (0.020) | (0.019) | (0.019) | (0.021) | (0.013) | (0.013) | (0.015) | (0.011) | (0.014) |
| retired | -0.051*** | -0.129*** | -0.038* | -0.023 | 0.006 | 0.004 | 0.006 | 0.023* | 0.006 | -0.009 |
|  | (0.015) | (0.020) | (0.021) | (0.025) | (0.024) | (0.014) | (0.014) | (0.013) | (0.012) | (0.013) |
| student | -0.057*** | -0.159*** | -0.167*** | -0.197*** | -0.174*** | -0.007 | 0.021 | 0.042*** | 0.006 | 0.004 |
|  | (0.015) | (0.029) | (0.042) | (0.053) | (0.054) | (0.016) | (0.015) | (0.009) | (0.013) | (0.013) |
| housekeeper | -0.064*** | -0.122*** | -0.098*** | -0.045 | -0.122*** | -0.043 | -0.009 | -0.006 | -0.012 | -0.025 |
|  | (0.014) | (0.027) | (0.025) | (0.029) | (0.040) | (0.034) | (0.029) | (0.026) | (0.020) | (0.024) |
| other | -0.032 | -0.124* | -0.224*** | -0.004 | -0.011 | -0.080 | -0.000 | -0.074 | -0.056 |  |
|  | (0.106) | (0.063) | (0.068) | (0.059) | (0.055) | (0.068) | (0.041) | (0.058) | (0.058) |  |
| foreign nationality | -0.012 | -0.043 | -0.045 | 0.000 | -0.043 | -0.018 | -0.017 | -0.027* | -0.015 | -0.011 |
|  | (0.025) | (0.045) | (0.054) | (0.034) | (0.031) | (0.014) | (0.018) | (0.014) | (0.012) | (0.013) |
| Religion |  |  |  |  |  |  |  |  |  |  |
| (ref: catholic) |  |  |  |  |  |  |  |  |  |  |
| other religion | -0.027 | -0.056 | -0.052 | -0.045 | -0.026 | -0.106*** | -0.128*** | -0.142*** | -0.052** | -0.064** |
|  | (0.022) | (0.035) | (0.036) | (0.037) | (0.038) | (0.035) | (0.032) | (0.045) | (0.025) | (0.028) |
| atheist/agnostic | -0.013 | -0.006 | 0.007 | -0.004 | -0.021* | -0.010 | -0.016* | -0.016* | -0.015** | -0.013* |
|  | (0.008) | (0.014) | (0.014) | (0.014) | (0.013) | (0.009) | (0.009) | (0.009) | (0.007) | (0.008) |
| Diagnosed COVID-19 | 0.003 | 0.019 | 0.052** | -0.113*** | -0.177*** | -0.102*** | -0.067*** | -0.045*** | 0.000 | -0.018* |
|  | (0.015) | (0.024) | (0.026) | (0.027) | (0.027) | (0.018) | (0.018) | (0.017) | (0.010) | (0.010) |
| Hospitalised COVID-19 | 0.060 | -0.075* | -0.125** | -0.075 | -0.024 | -0.079 | 0.002 | -0.004 | -0.020 | -0.055 |
|  | (0.061) | (0.043) | (0.056) | (0.067) | (0.073) | (0.058) | (0.035) | (0.043) | (0.035) | (0.065) |
| Region FE | Yes | Yes | Yes | Yes | Yes | Yes | Yes | Yes | Yes | Yes |
|  |  |  |  |  |  |  |  |  |  |  |
| Observations | 3,669 | 3,713 | 3,689 | 3,668 | 3,662 | 3,633 | 3,522 | 3,518 | 3,584 | 3,609 |
| NOTES: Each column reports coefficients from a different logit regression where the dependent variable is vaccination status at each month. Robust standard errors in parentheses. *** p<0.01, ** p<0.05, * p<0.1. | | | | | | | | | | |

Figure A4- Robustness test 1-a. Marginal effects on the probability of being vaccine hesitant by current political affiliation.


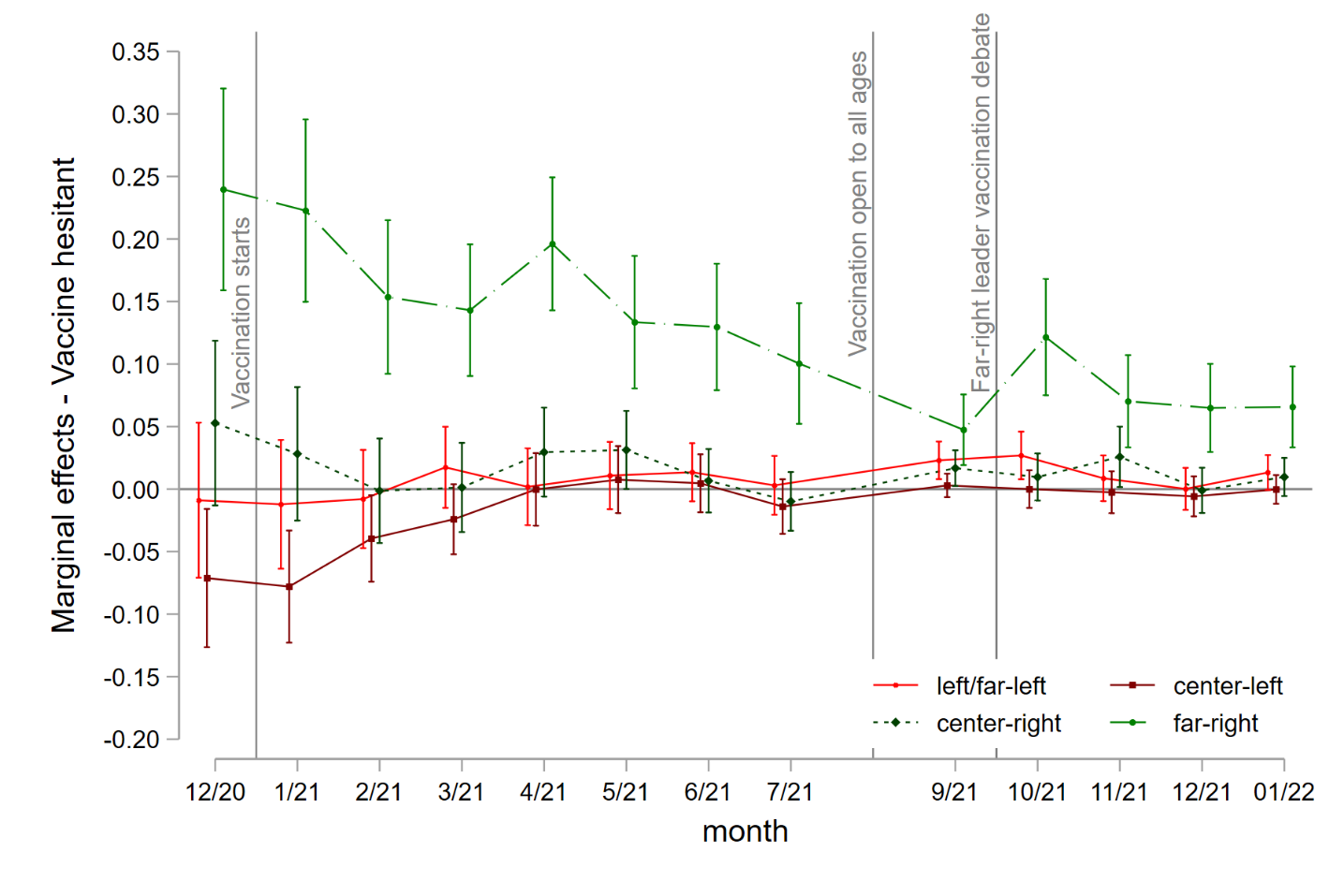


Notes: Coefficients for each month come from a different regression. Marginal effects (with 95% confidence intervals) from logit regressions fully adjusted by age, sex, education level, marital status, employment status, nationality, religion and region fixed effects. Political affiliation is based on the party they chose in the following *voting intention* question: “Imagine that tomorrow there are new general elections, which party would you vote for?” To those who declared not to vote, or “don’t know to” in the previous question, we assign them to the party that they chose in the following sympathy question: “Would you tell me towards which party you feel more sympathy?”. Then, we group them into ideological groups following Table A2. The remaining ones were grouped in the group “no voting or no response”. The reference category is centre.

Figure A5- Robustness test 1-b. Marginal effects on the probability of being vaccinated by current political affiliation.


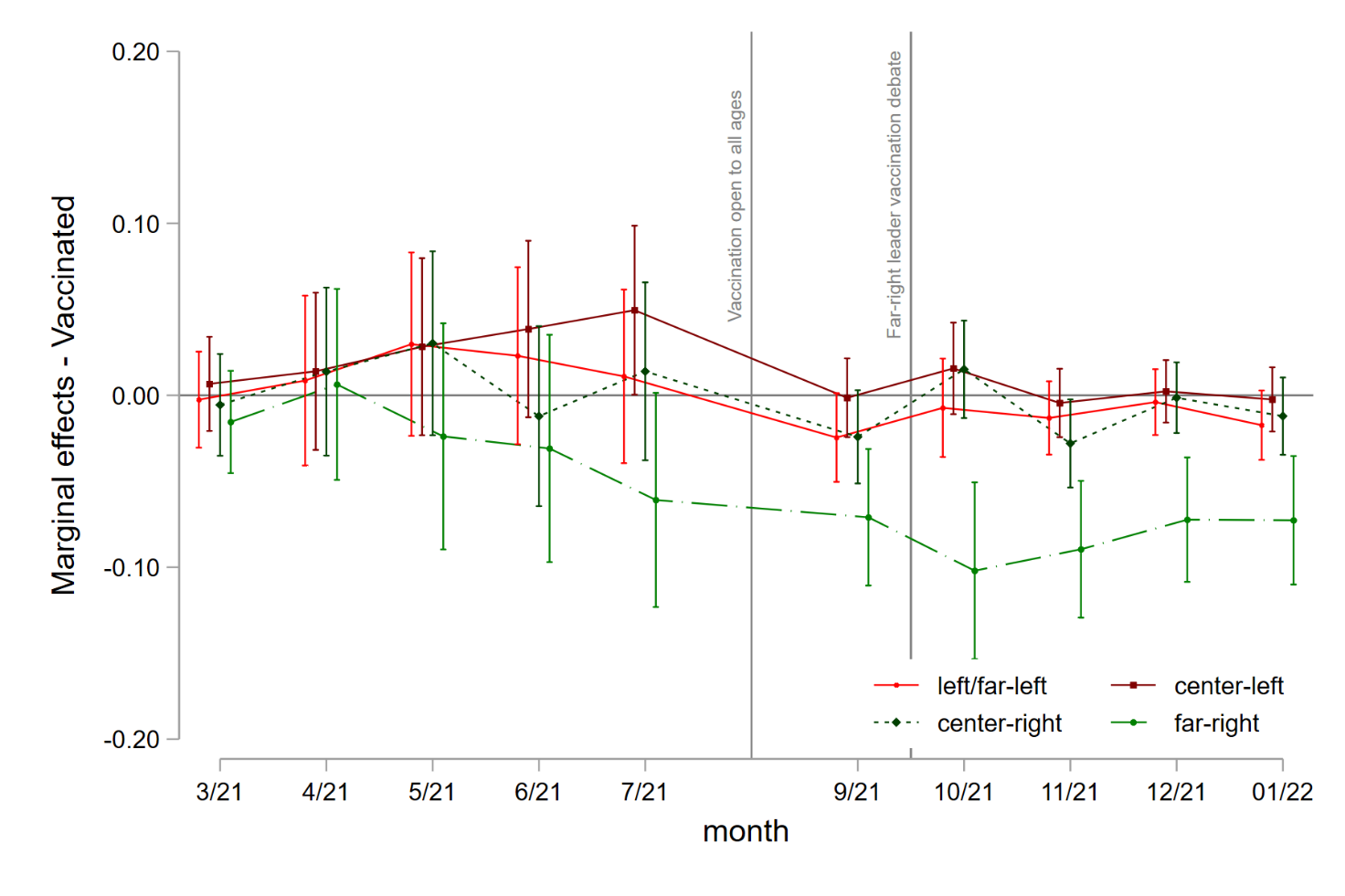


Notes: Coefficients for each month come from a different regression. Marginal effects (with 95% confidence intervals) from logit regressions fully adjusted by age, sex, education level, marital status, employment status, nationality, religion and region fixed effects. Political affiliation is based on the party they chose in the following *voting intention* question: “Imagine that tomorrow there are new general elections, which party would you vote for?” To those who declared not to vote, or “don’t know to” in the previous question, we assign them to the party that they chose in the following sympathy question: “Would you tell me towards which party you feel more sympathy?”. Then, we group them into ideological groups following Table A2. The remaining ones were grouped in the group “no voting or no response”. The reference category is centre.

Figure A6 - Robustness test 2. Evolution of vaccine hesitancy in regions that implemented


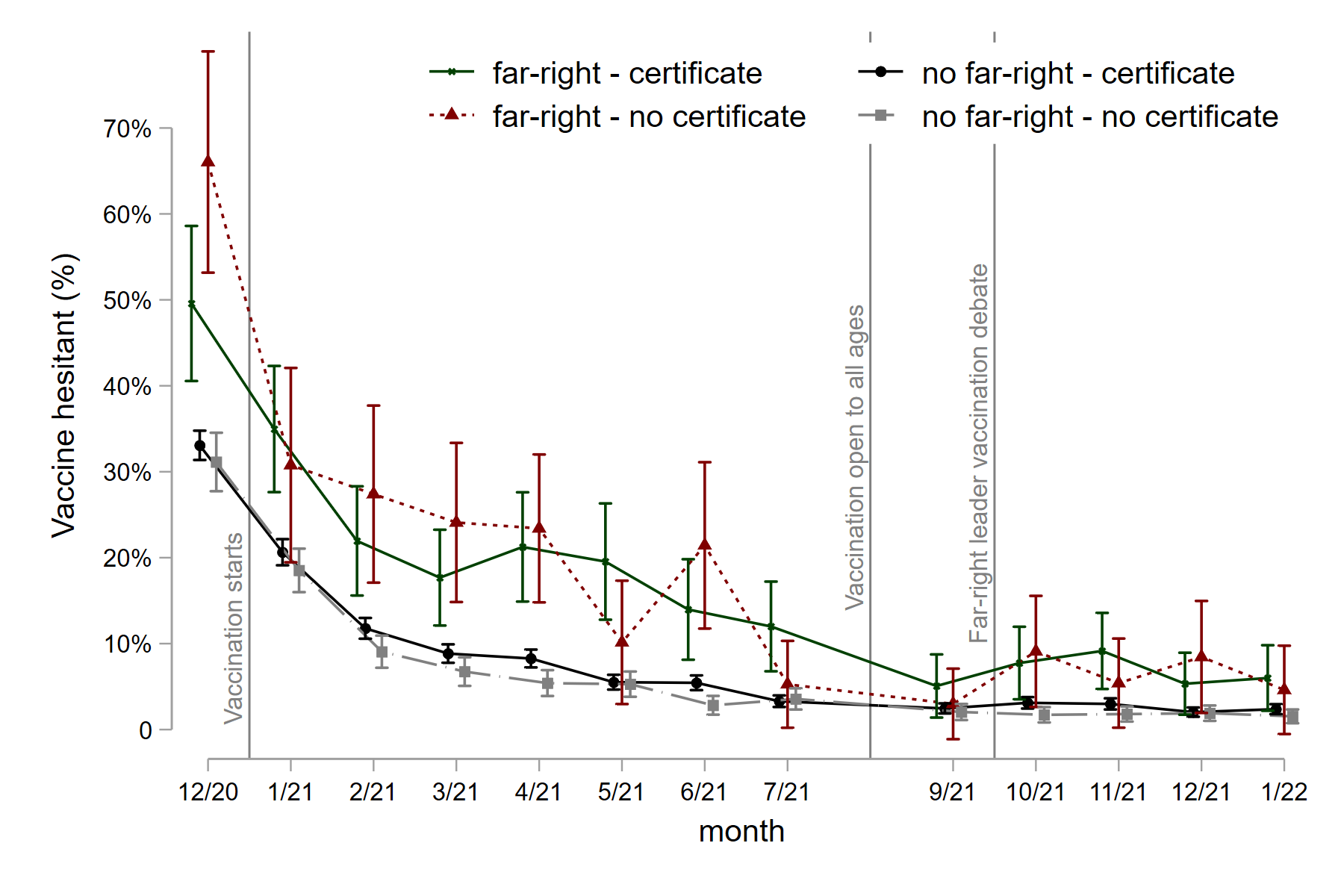


NOTES: This figure divides the pooled sample into 4 groups: i) “far-right – certificate”, those who voted for far-right from regions who implemented a COVID-19 certificate (n= 1,968); ii) “far-right – no certificate”, those who voted for far-right from regions who never implemented a COVID-19 certificate (n= 937); iii) “no far-right –certificate”, those who did not vote for far-right from regions who implemented a COVID-19 certificate (n= 35,134); iv) “no far-right – no certificate”, those who did not vote for far-right from regions who never implemented a COVID-19 certificate (11,306). The regions that implemented a COVID-19 certificate (by month of implementation) were the following: Baleares, Cataluña (October); Galicia, Murcia (November); Andalucia, Aragón, Canarias, Cantabria, Comunidad Valenciana, Navarra, Pais Vasco, Melilla (December): Asturias, Ceuta (January). The regions that did not implement a COVID-19 certificate were the following: Castilla – La Mancha, Castilla y León, Extremadura and Madrid.
